# Contribution of front-line, standard-of-care drugs to bactericidal responses, resistance emergence, and cure in murine models of easy- or hard-to-treat tuberculosis disease

**DOI:** 10.1128/aac.01901-24

**Published:** 2025-03-26

**Authors:** Nathan Peroutka-Bigus, Elizabeth J. Brooks, Michelle E. Ramey, Hope D'Erasmo, Jackie P. Ernest, Allison A. Bauman, Lisa K. Woolhiser, Radojka M. Savic, Anne J. Lenaerts, Bree B. Aldridge, Jansy P. Sarathy, Gregory T. Robertson

**Affiliations:** 1 Mycobacteria Research Laboratories, Department of Microbiology, Immunology and Pathology, Colorado State University 3447, Fort Collins, Colorado, USA; 2 Department of Molecular Biology and Microbiology, Tufts University School of Medicine 12261, Boston, Massachusetts, USA; 3 Department of Bioengineering and Therapeutic Sciences, University of California San Francisco 8785, San Francisco, California, USA; 4 The Stuart B. Levy Center for Integrated Management of Antimicrobial Resistance, Boston, Massachusetts, USA; 5 Department of Biomedical Engineering, Tufts University School of Engineering 98044, Medford, Massachusetts, USA; 6 Center for Discovery and Innovation, Hackensack Meridian Health, Nutley, New Jersey, USA; 7 Department of Medical Sciences, Hackensack Meridian School of Medicine 576909 , Nutley, New Jersey, USA; St. George's, University of London, London, United Kingdom

**Keywords:** tuberculosis, relapse, caseum, rifafour, C3HeB/FeJ

## Abstract

By assessing the standard-of-care regimen for tuberculosis (TB) in BALB/c and C3HeB/FeJ mice, we demonstrate that rifampin, with or without pyrazinamide, is essential for an effective bactericidal response and suppression of resistance. Potency measurements in an *in vitro* lipid-rich model and a rabbit caseum assay recapitulate the significance of rifampin as a sterilizing agent. These outcomes align with clinical performance, thus emphasizing the value of *in vitro* predictive tools and murine TB models with human-like pathology.

## INTRODUCTION

Fox described human clinical studies that led to the current standard-of-care chemotherapy for tuberculosis (1). Rifampin (R) and pyrazinamide (Z) were denoted as key sterilizing drugs, with isoniazid (H) contributing to bactericidal responses, while ethambutol (E), a bacteriostatic drug, was described as contributing little to bactericidal responses or sterilizing cure (1).

**Fig 1:**
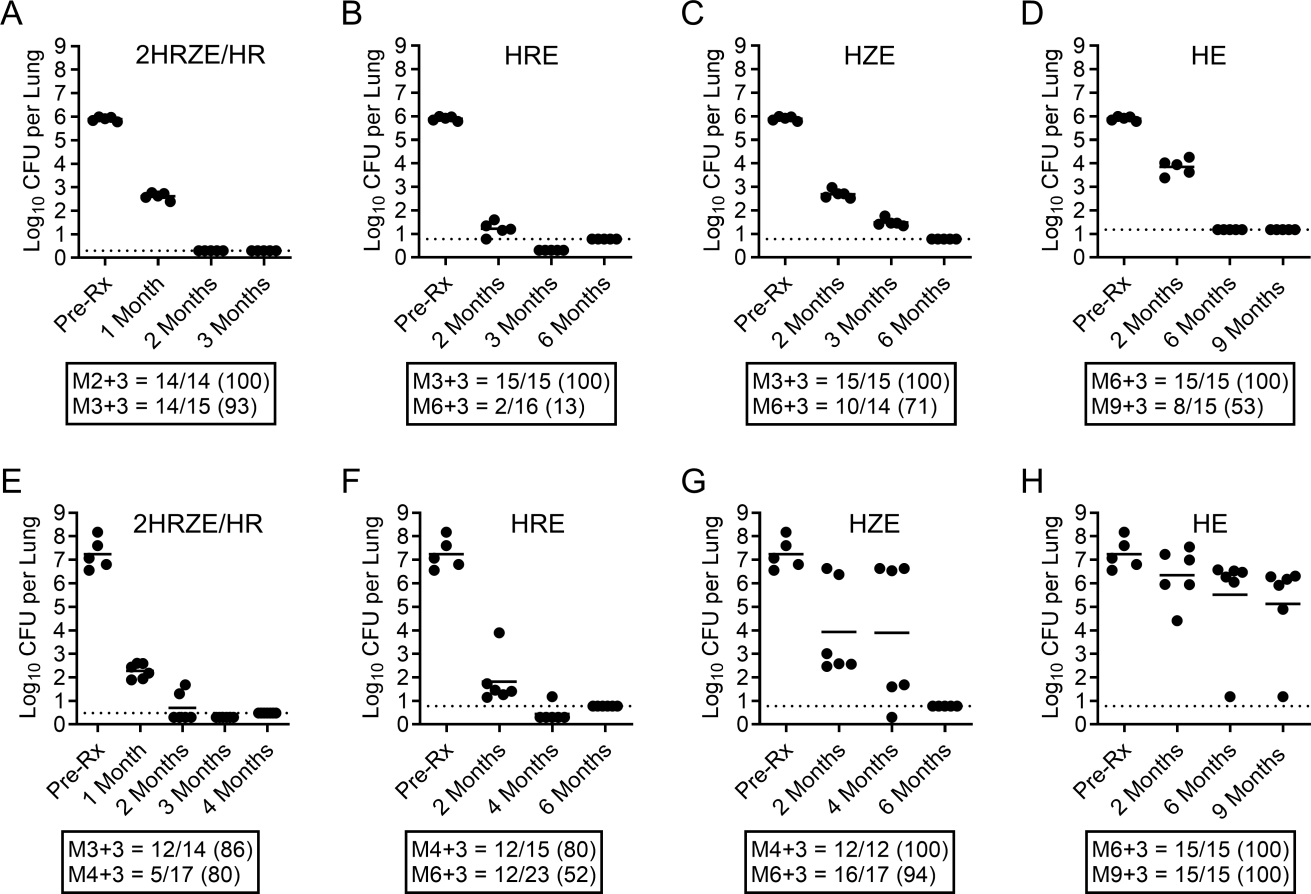
*Mycobacterium tuberculosis* Erdman CFU burdens in the lungs of BALB/c (A–D) and C3HeB/FeJ (E–H) mice at the start of treatment (Pre-Rx) or after treatment for the indicated number of months with 2HRZE/HR (A and E), HRE (B and F), HZE (C and G), or HE (D and H). Closed circles represent individual mice. The solid horizontal line is the group mean value. The dashed horizontal line is the upper lower limit of detection; for points that fall beneath this dashed line, animals had lower limits of detection due to variation in CFU plating. The box beneath represents the number of mice that relapsed 3 months after the indicated treatment duration over the group total; the proportion of mice relapsing is listed in parentheses. Treatment durations and experimental time points were chosen based upon previous research with these two mouse models; as such, there are instances where matched time points were not taken.

Using two pathologically distinct murine tuberculosis (TB) models, we sought to interrogate treatment outcomes for the standard-of-care regimen (see Methods in supplemental material). In BALB/c mice chronically infected with *Mycobacterium tuberculosi*s (Mtb) Erdman (without the caseating granulomas seen in patients [2]), HRZE reduced BALB/c lung burdens by 3.28 logs after 1 month of treatment, by 5.60 logs after 2 months (three out of five mice remained culture positive), and returned no CFU after 3 months of 2HRZE/HR (Fig. 1A; Table S1), indicating potent bactericidal responses. HRZE was equally potent in C3HeB/FeJ mice chronically infected with Mtb Erdman (showing more human-like, heterogeneous lung pathology [2–4]), reducing lung burdens by 4.97 logs after 1 month of treatment, by 6.54 logs after 2 months (four out of six mice remained culture positive) (Fig. 1E; Table S1), and returned no CFU after 3 months of 2HRZE/HR in C3HeB/FeJ except for one mouse returning a single CFU. These results agree with previously published findings (5).

HRE, HZE, and HE showed to be less rapidly bactericidal vs HRZE in BALB/c mice, promoting 4.69, 3.21, and 2.06 log10 CFU reductions in lungs, respectively, after 2 months of treatment (highest *P* value = 0.0001). No CFUs were cultured after 6 months of HRE or HZE, and only one of five mice in the HE group was culture positive. All HE-treated mice were culture negative by month 9 (Fig. 1B through D; Table S1). Similar to the BALB/c arm, HRE reduced C3HeB/FeJ lung burdens by 5.43 logs after 2 months of treatment (Fig. 1F; Table S1), revealing a minor role for Z in the HRZE bactericidal response. However, substitution of Z for R in HZE (HZE vs HRE, *P* = 0.0482) or adding R or Z to HE (HE vs HRE, *P* < 0.0001; HE vs HZE, *P* = 0.0214) improved efficacy significantly (3.31 and 0.89 logs from the start of treatment, respectively) (Fig. 1F through H; Table S1). No CFUs were cultured after 6 months of HRE or HZE. However, all mice in the HE group were culture positive following 6 and 9 months of treatment (Fig. 1H; Table S1). Interestingly, a bimodal response was seen in C3HeB/FeJ mice administered regimens lacking R or RZ, whereby a subpopulation of mice was less responsive to drug treatment based on pulmonary pathology (6–8). This was not similarly observed in the BALB/c arm. Only in combinations including R or RZ, was a more effective and uniform treatment response observed in C3HeB/FeJ mice, similar to the BALB/c arm (compare Fig. 1A and B to Fig. 1E and F). As described by Fox for human TB patients (1), more mice underwent relapse following 6 months of treatment with regimens lacking R or RZ (Fig. 1B through D and Fig. 1F through H). HE was the least effective in C3HeB/FeJ mice, with all mice relapsing following 9 months of treatment (Fig. 1H; Table S2A and B).

Co-plating on antibiotic-containing agar plates after 2HRZE/HR therapy in C3HeB/FeJ mice resulted in rare resistant isolates to H, R, or E (see Table S3A), similar to the HRE or HZE groups. In contrast, five of the six C3HeB/FeJ mice treated with HE had a high number of isolates that grew on 0.2 mg/L of H, with a low frequency of resistance to E, suggesting that H resistance occurred without loss of susceptibility to E in many cases (see Table S3A). Higher rates of resistance were observed in the C3HeB/FeJ study arm during relapse, which was far lower in the BALB/c arm (see Table S3B and S4B). Further studies are necessary to understand the biological basis of this outcome.

**Fig 2:**
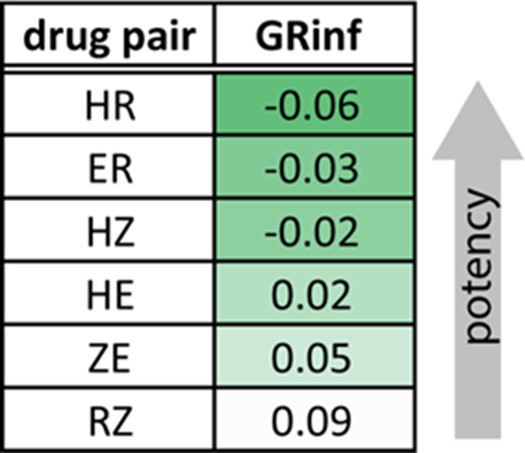
Pairwise drug combination potencies in medium with butyrate as the sole carbon source measured as GRinf after 6 days of treatment using diagonal measurement of n-way drug interactions (DiaMOND). Data are derived from Larkins-Ford et al. (9).

To further investigate why R was a good partner drug *in vivo*, we systematically evaluated *in vitro* drug combination effects of six drug pairs from HRZE using data from Larkins-Ford et al. (9). Pairwise potencies were evaluated in a lipid-rich environment (butyrate), where Z shows activity, using the infinite growth rate (GRinf), a metric of the combination potency and predictor of relapsing outcomes (9). HR and RE were the most potent and more potent than HZ and ZE (Fig. 2). Together, these data suggested that R pairs well with E and H in lipid-rich environments.

**Fig 3:**
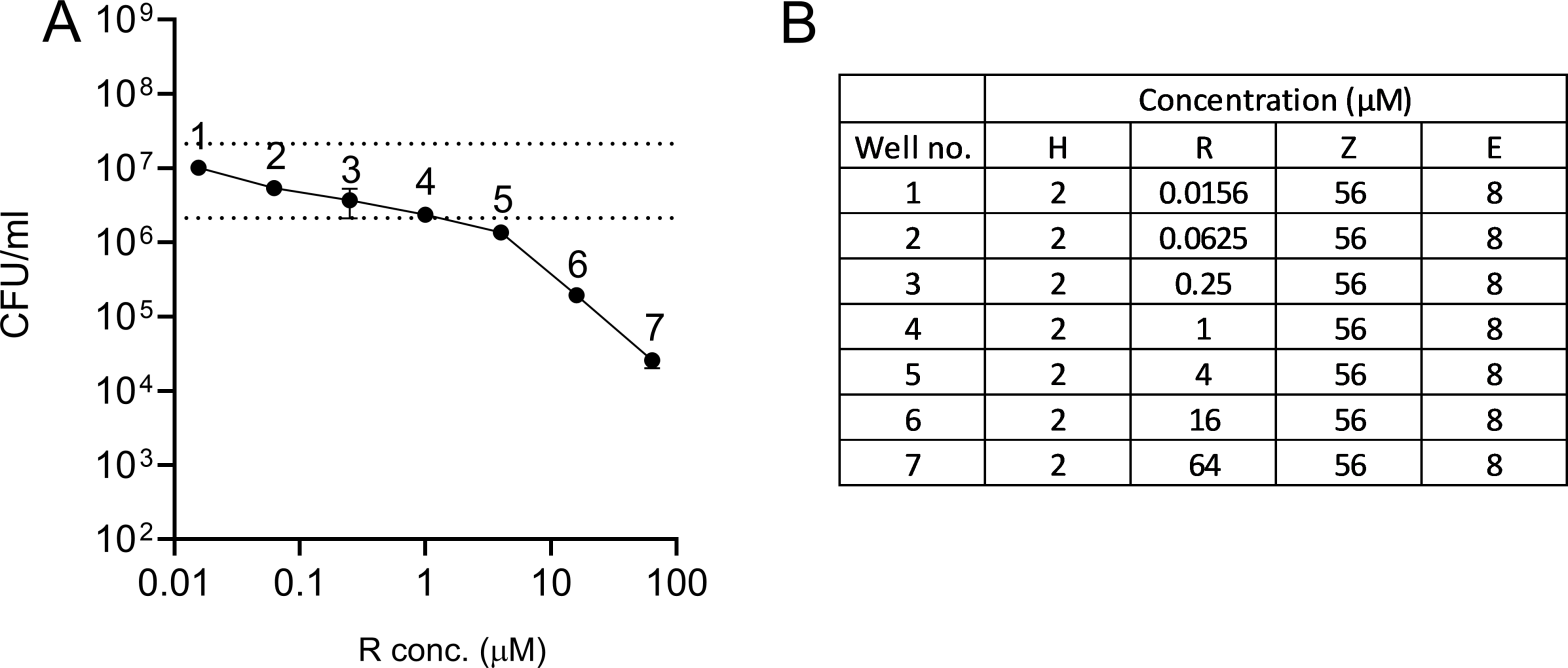
Bactericidal activity of HRZE in *ex vivo* rabbit caseum (A). Bacterial burden and R concentration are expressed on log scales. The dotted line indicates the bacterial burden in the DMSO-only control well and the cutoff for a 1-log reduction. Concentrations used for each drug at each data point are shown in the inset table (B). Data points and error bars represent the means and standard deviations for three technical replicates each.

HRZE was examined in *ex vivo* rabbit caseum (Fig. 3), evaluating drug potency against non-replicating bacteria (10). Previously, H, Z, and E were shown to be minimally active (casMBC_90_s > 512 µM). Only R produced significant killing (casMBC_90_ of 10 µM) (11, 12). Here, R concentrations were tested centered around 4 µM (0.0156–64 µM) to approximate the average concentrations in caseum (C_ave[0–24]_). H, Z, and E were held static at 2, 56, and 8 µM, their respective caseum C_ave[0–24]_s (Fig. 3B). Increased R exposure in HRZE produced increased bacterial killing in rabbit caseum, achieving 1-log killing when all drugs were present at C_ave[0–24]_ (data point 5) (Fig. 3A). This result suggests a role for the potency of R against nonreplicating Mtb from caseous granulomas in driving treatment efficacy in C3HeB/FeJ mice.

In conclusion, while 2HRZE/HR and HRE showed nearly identical activities in BALB/c and C3HeB/FeJ mice, regimens lacking R or RZ were far less efficacious in C3HeB/FeJ mice in terms of bactericidal response, prevention of relapse, and suppression of resistance emergence. These results align well with the observations by Fox (1) and with the data reported in 8-week early bactericidal activity trials (13), highlighting the contribution of R or RZ to the current front-line TB regimen (14). High rates of resistance were noted for C3HeB/FeJ mice given only HE but not for mice on Z- or RZ-containing regimens. It is not yet clear if this reflects regional differences in local drug exposures (i.e., pockets of regional monotherapy) or differences in bacterial phenotype in lesions imparting reduced susceptibility to E. Fox similarly reported a minor role for E in initial resistance to H and suppression of R resistance in cases where the infection is resistant to H (1). Collectively, this work illustrates the ability of diverse murine TB efficacy models of increasing complexity to better highlight differences in regimen behavior(s) and assessing contribution of individual drugs to regimens. The results also highlight the potential of new systematic *in vitro* approaches in TB drug development using conditions that reproduce lesion microenvironments to develop predictive classifiers of multidrug treatment outcomes (6), providing a rationale for the prioritization of combinations to take forward for resource-intensive *in vivo* testing. The data also reveal two strengths of the C3HeB/FeJ chronic TB mouse model in pre-clinical testing absent from the BALB/c model: (i) to evaluate the *in vivo* potential for drug resistance emergence in a model with heterogeneous lesion pathology, and (ii) the ability to identify drugs and drug combinations with differential treatment responses based on complex lesion pathology (bimodal response seen in less efficacious regimens). Bimodal responses to drug treatment in this model have been attributed to limited necrotic lung disease in responders vs appreciable necrotic lung disease in low responders (6, 8). Future studies will, therefore, benefit from qualitative gross pathology scores at the time of tissue collection to help correlate the extent of pulmonary disease with treatment outcomes.

## SUPPLEMENTAL MATERIAL

Supplemental material aac.01901-24-s0001.docx Tables S1 to S4 and supplemental methods.

ASM does not own the copyrights to Supplemental Material that may be linked to, or accessed through, an article. The authors have granted ASM a non-exclusive, world-wide license to publish the Supplemental Material files. Please contact the corresponding author directly for reuse.
