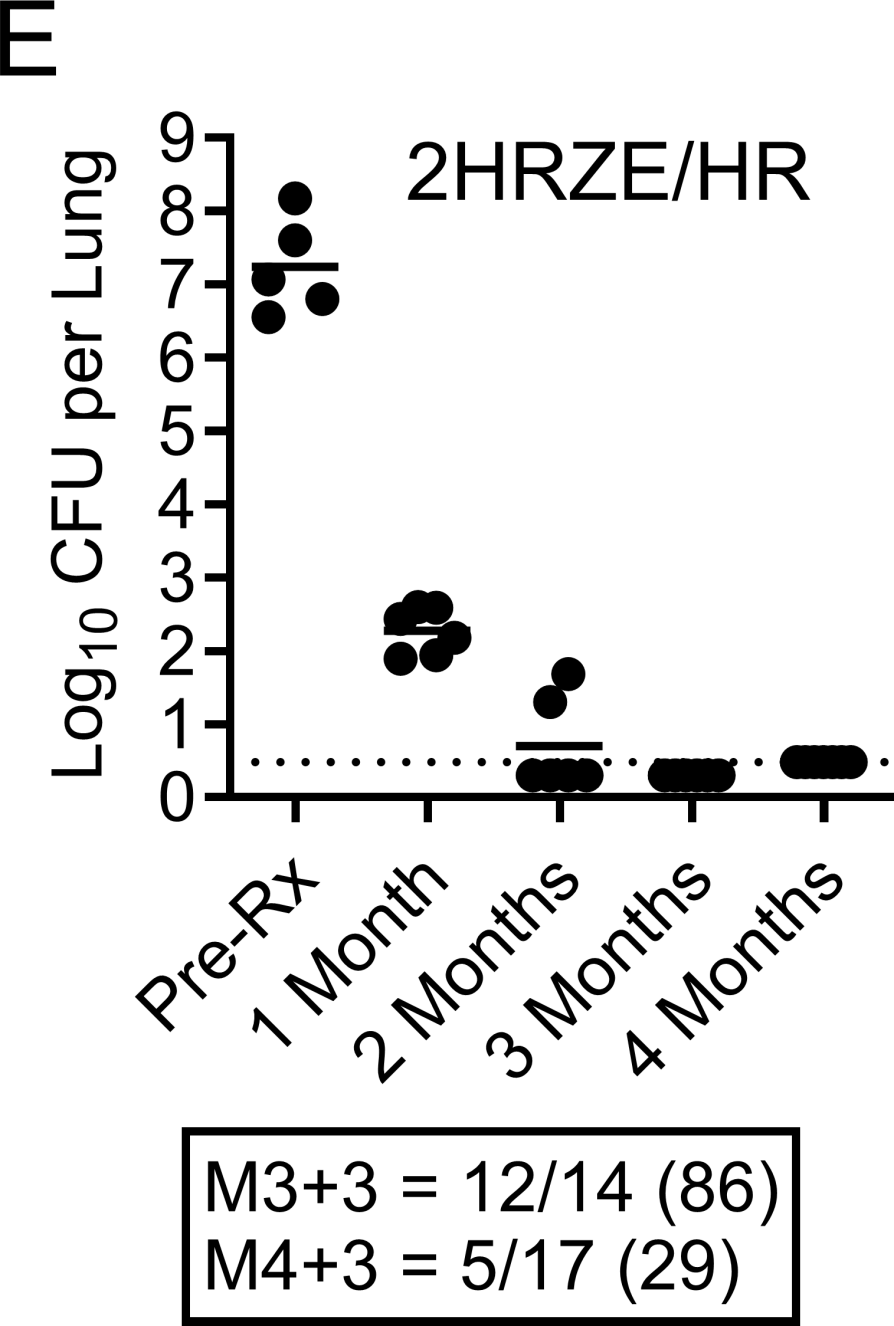# Erratum for Peroutka-Bigus et al., “Contribution of front-line, standard-of-care drugs to bactericidal responses, resistance emergence, and cure in murine models of easy- or hard-to-treat tuberculosis disease”

**DOI:** 10.1128/aac.00654-25

**Published:** 2025-06-26

**Authors:** Nathan Peroutka-Bigus, Elizabeth J. Brooks, Michelle E. Ramey, Hope D’Erasmo, Jackie P. Ernest, Allison A. Bauman, Lisa K. Woolhiser, Radojka M. Savic, Anne J. Lenaerts, Bree B. Aldridge, Jansy P. Sarathy, Gregory T. Robertson

## ERRATUM

Volume 69, no. 5, e01901-24, 2025, https://doi.org/10.1128/aac.01901-24. Page 2: Fig. 1E should appear as shown in this erratum. The number of mice relapsing at M4 + 3 was entered correctly but the accompanying percentage was incorrect and has been updated. The erratum does not change the conclusions of the original publication.

**Fig 1:**