# Supplementary material for: Contribution of front-line, standard-of-care drugs to bactericidal responses, resistance emergence, and cure in murine models of easy- or hard-to-treat tuberculosis disease
Source: Antimicrob Agents Chemother. 2025 Mar 26;69(5):e01901-24. doi: 10.1128/aac.01901-24 (PMC12057366; doi:10.1128/aac.01901-24)
Supplement: Supplemental material — Tables S1 to S4 and supplemental methods. [file aac.01901-24-s0001.docx]

**Supplemental data**

**Title:** Contribution of front-line standard-of-care drugs to bactericidal responses, resistance emergence, and cure in murine models of easy- or hard-to-treat tuberculosis disease.

**Authors**

Nathan Peroutka-Bigus^1^, Elizabeth J. Brooks^1^, Michelle E. Ramey^1^, Hope D’Erasmo^2^, Jackie P. Ernest^3^, Allison A. Bauman^1^, Lisa K Woolhiser^1^, Radojka M. Savic^3^, Anne J. Lenaerts^1^, Bree B. Aldridge^2,4,5^, Jansy P. Sarathy^6,7^, and Gregory T. Robertson^1^

**Affiliation:**

^1^Mycobacteria Research Laboratories, Department of Microbiology, Immunology and Pathology, Colorado State University, Fort Collins, Colorado, USA.

^2^Department of Molecular Biology and Microbiology, Tufts University School of Medicine, Boston, Massachusetts, USA

^3^Department of Bioengineering and Therapeutic Sciences, University of California San Francisco, San Francisco, California, USA.

^4^The Stuart B. Levy Center for Integrated Management of Antimicrobial Resistance, Boston, Massachusetts, USA.

^5^Department of Biomedical Engineering, Tufts University School of Engineering, Medford, Massachusetts, USA.

^6^Center for Discovery and Innovation, Hackensack Meridian Health, Nutley, New Jersey, USA ^7^Hackensack Meridian School of Medicine, Department of Medical Sciences, Nutley, New Jersey, USA.

Corresponding Author: Gregory.Robertson@colostate.edu

**Keywords:** tuberculosis, relapse, caseum, rifafour, C3HeB/FeJ

**Suppl. Table 1.** *Mycobacterium tuberculosis* lung CFU counts assessed during treatment of BALB/c or C3HeB/FeJ mice. The numbers in brackets indicate the number of mice for which CFU were recovered over the total number of mice at the time of plating.

**Suppl. Table 2.** P values using the Fisher's exact test for the proportions of BALB/c (A) or C3HeB/FeJ (B) mice relapsing 3-months after 2HRZE/HR, HRE, HZE or HE for the indicated treatment time (in months).

**A**


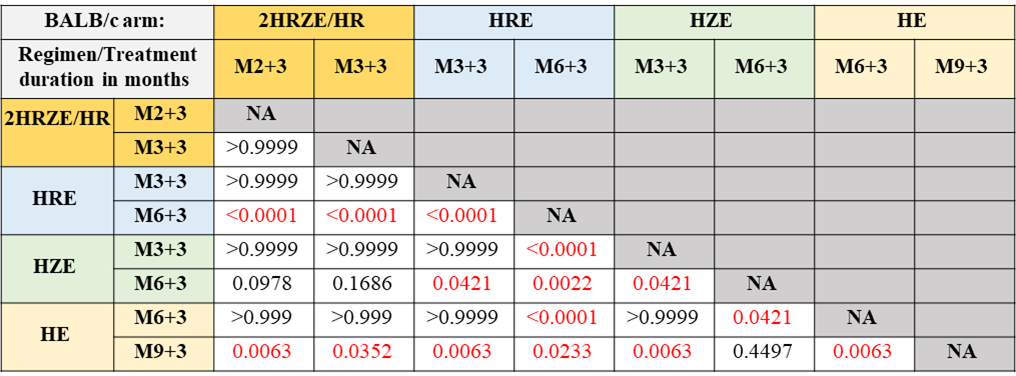


**B**


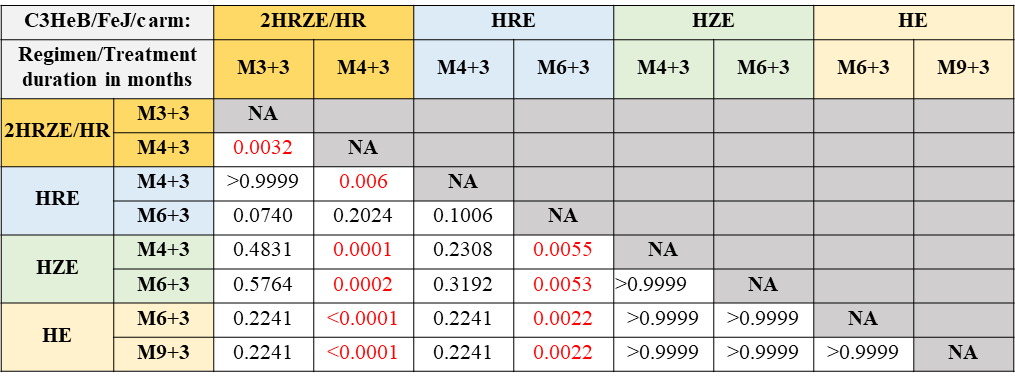


**Suppl. Table 3**. Emergence of drug-resistant *Mycobacterium tuberculosis* in the C3HeB/FeJ study arm for the indicated treatment groups at the end of each treatment arm (A) or following a 3-month drug-free relapse period (B).

**A**


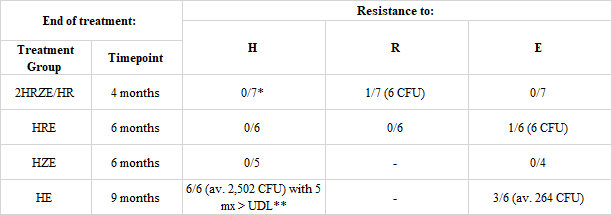


**B**


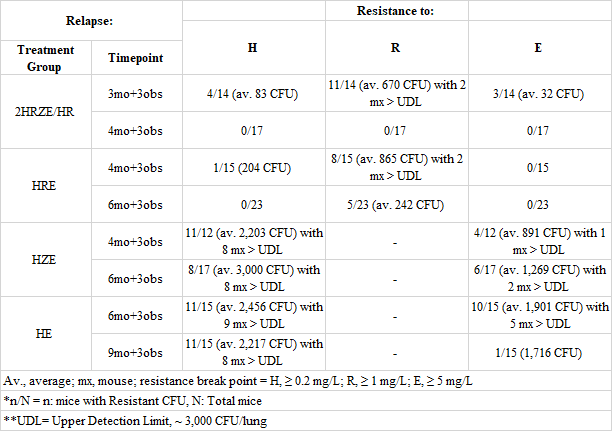


**Suppl. Table 4**. Emergence of drug-resistant *Mycobacterium tuberculosis* in the BALB/c study arm for the indicated treatment groups at the end of each treatment arm (A) or following a 3-month drug-free relapse period (B).

**A**


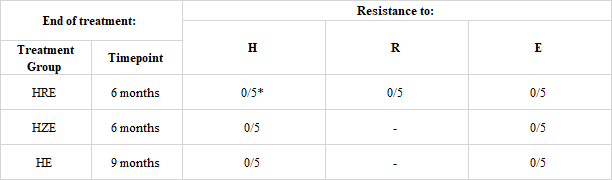


**B**


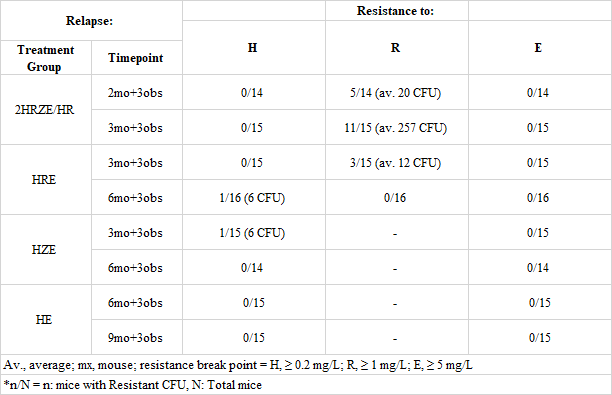


**Methods**

**Murine Infection Models.***M. tuberculosis* strain Erdman (TMCC 107) was used in these infection studies. The Colorado State University Institutional Animal Care and Use Committee (IACUC) (Reference numbers of approved protocol: KP 1515) approved all procedures used herein. Mice were housed in an ABSL3 facility and were provided *ad libitum* access to food and water. Aerosol infection was performed using a Glas-Col inhalation exposure system using previously calibrated frozen stocks of *Mtb* Erdman. For both the BALB/c and C3HeB/FeJ infection studies, 8- to 10-week-old mice were obtained from Jackson Laboratories (Bar Harbor, ME). One day after the low-dose aerosol infection a subset of mice were sacrificed to determine the initial bacterial burden in the lungs and was found to be approximately 2.21 log10 CFU (~162 CFU) in BALB/c mice and 1.55 log10 CFU (35 CFU) for C3HeB/FeJ mice.  Mice exhibiting clinical symptoms of illness were humanely euthanized.

**Antimicrobial preparation and administration to mice.**Rifampin (R), isoniazid (H), and ethambutol (E) were purchased from Sigma, while Pyrazinamide (Z) was purchased from Acros Organics. All drugs were prepared in sterile water at 1× to 3× their respective concentration needed to achieve a final dose of 10 mg/kg (R), 10 mg/kg (H), 150 mg/kg (Z), and 100 mg/kg (E). Z was briefly heated to 60°C until fully dissolved and R was ground to a fine powder with a mortar and pestle. Drugs were prepared in weekly batches and kept at 4℃ and aliquoted for daily use. Drugs were administered by oral gavage once daily and given 5 days per week (Monday through Friday). R was administered individually in a 0.2 mL volume, followed >2 h later by any other drug combination. HZ, HE, or HZE, were combined immediately before administration and given in a single 0.2 mL volume.

**Drug efficacy experiments and bacterial enumeration.** Drug efficacy determinations were based on lung CFU counts from whole lungs aseptically harvested 3-days after the last day of dosing to allow drug clearance from tissues, or 3 months following the last day of dosing for the relapse arm of the study. Tissues were homogenized (Glas-Col Tissue Homogenizer, Terre Haute, IN) and serially diluted. Portions of these homogenates were plated for CFU on 7H11-OADC agar (i.e., Middlebrook 7H11 agar plates supplemented 0.2% [v:v] glycerol, 10% [v:v] oleic acid-albumin-dextrose-catalase (OADC) supplement, and 0.01 mg/mL cycloheximide, and 0.05 mg/mL carbenicillin). Colonies were enumerated after at least 28 days of incubation at 37°C and plates were incubated for ≥ 6 weeks to ensure all viable colonies were detected. Mice were euthanized by CO_2_ inhalation followed by cervical dislocation, a method approved by the IACUC at Colorado State University.

**Statistical analysis***.* The viable CFU counts per organ were log transformed and evaluated by a one-way analysis of variance (ANOVA) with multiple comparison using either Tukey’s test (pairwise comparison between all treatment groups) or Dunnett’s test (for comparison of each treatment to the start of treatment controls) using Prism 10 (GraphPad Software, San Diego, CA). Differences in relapse proportions were assessed by Fisher’s Exact test using the Holm-Bonferroni correction for multiple comparisons. Differences were considered significant at the 95% level of confidence.

**Caseum MBC assay**. The bactericidal activity of H, R, Z and E in combination against nonreplicating *Mtb* in rabbit caseum was conducted as described previously (1, 2). Briefly, wells of a 96-well plate were spotted with all four drugs to achieve the final test concentrations in Fig 3. Overall, the dose-response curve was designed with 4-fold increments in RIF concentrations, while keeping H, Z and E static on average concentrations achieved in the caseous compartments of lesions in patients receiving standard dosing regimens. 50 µl of 3-fold diluted caseum homogenate was added to each well in 96-well plates, which were then incubated at 37°C for 7 days. After incubation, each well was sampled and serially diluted in PBS containing 0.0125% Tween 80 prior to plating on 7H11 agar plates. Colony-forming units (CFU) were enumerated ≥ 3 weeks later.

**References**

1. Sarathy JP, Via LE, Weiner D, Blanc L, Boshoff H, Eugenin EA, Barry CE III, Dartois VA. 2018. Extreme drug tolerance of *Mycobacterium tuberculosis* in caseum. *Antimicrob Agents Chemother* 62:e02266-17. https://doi.org/10.1128/AAC.02266-17

2. Sarathy JP, Xie M, Jones RM, Chang A, Osiecki P, Weiner D, Tsao W-S, Dougher M, Blanc L, Fotouhi N, Via LE, Barry CE III, De Vlaminck I, Sherman DR, Dartois VA. 2023. A novel tool to identify bactericidal compounds against vulnerable targets in drug-tolerant *M. tuberculosis* found in caseum. *mBio* 14:e0059823. https://doi.org/10.1128/mbio.00598-23
